# COVID-19 and Alcohol Poisoning: A Fatal Competition

**DOI:** 10.1017/dmp.2021.89

**Published:** 2021-03-25

**Authors:** Mohammad Heidari, Nasrin Sayfouri

**Affiliations:** 1 Community-Oriented Nursing Midwifery Research Center, Shahrekord University of Medical Sciences, Shahrekord, Iran; 2 School of Health Management and Information Sciences, Iran University of Medical Sciences, Tehran, Iran

**Keywords:** COVID-19, coronavirus, alcohol consumption, alcohol poisoning

## Abstract

In March 2020, concurrently with the outbreak of coronavirus disease 2019 (COVID-19) in Iran, the rate of alcohol poisoning was unexpectedly increased in the country. This study has attempted to make an overall description and analysis of this phenomenon by collecting credible data from the field, news, and reports published by the emergency centers and the Iranian Ministry of Health. The investigations showed that in May 20, 2020, more than 6150 people have been affected by methanol poisoning from whom 804 deaths have been reported. A major cause of the increased rate of alcohol poisoning in this period was actually the illusion that alcohol could eliminate the coronaviruses having entered the body. It is of utmost importance that all mass media try to dismiss the cultural, religious, and political considerations and prepare convincing programs to openly discuss the side-effects of forged alcohol consumption with the public, especially with the youth. It must be clearly specified that “consuming alcohol cannot help prevent COVID-19.”

Alcohol poisoning is a preventable cause of some health problems in most communities. Ethanol and methanol are 2 major types of alcohol.^1,2^ However, due to the availability of methanol, a health-threatening type of alcohol, recent years have witnessed a rise in morbidity and mortality due to alcohol poisoning in Iran and some other low-income Muslim countries,^3,4^ to the extent that it has turned into one of the major medical and legal issues, particularly in Iran. Although there are plenty of studies reporting that alcohol consumption is widespread in some communities, in Iran, due to legal, religious, and political reasons, there is not adequate evidence-based information on this phenomenon.^5^ Generally, in Muslim countries, because of the prohibition of alcohol production, distribution, and consumption, poisoning with clandestinely produced alcohol and particularly methanol is increasing, especially among the youth in Iran.^4,6,7^ This study, being a part of a larger ongoing review study, has attempted to describe and analyze the phenomenon of the unprecedented morbidity and mortality due to alcohol poisoning occurred during March and April 2020 by collecting credible data from the field, news, and reports, the emergency centers, the Iranian Ministry of Health, and some published articles.

## Alcohol Poisoning in Iran

After the emergence of novel coronavirus in Wuhan, China, its related disease, called coronavirus disease 2019 (COVID-19) by the World Health Organization (WHO), was quickly expanded throughout the world.^8,9^ Iran was 1 of the first countries being involved with COVID-19. Upon the COVID-19 outbreak in Iran and increased fear and stress in the public, demands for sanitation-related items, including alcohol-based sanitizers and protective items (eg, face masks) increased dramatically resulting in scarcity of these items in the market. As a result, people turned to more available disinfectants, such as ethanol, as an alternative. Despite the recommendations that accurate hand washing is the easiest and the most efficient preventive measure, people continued using alcohol as the major disinfectant. Upon the increased demand for ethanol, some profiteers simply changed the appearance of methanol and sold it under the name of ethanol.

A short while after the onset of COVID-19 epidemics in Iran (in March 2020), alcohol poisoning became another emergency condition that expanded along with COVID-19. The number of cases of alcohol poisoning increased so dramatically in some Iranian regions that its mortality rate exceeded that of COVID-19. All Iranian provinces reported methanol poisoning cases while 26 of 31 provinces reported deaths.^10^ Between March and April 2020, the largest methanol mass poisoning outbreak occurred, which was unprecedented in Iran and the whole world in recent decades.^11^ This event accounted for 924 and 998 morbidity cases of alcohol poisoning and 104 and 79 mortality cases in Fars and Khuzestan Provinces, respectively, since the onset of COVID-19. These numbers are higher than those associated with COVID-19 in the same provinces (as of May 20). More importantly, the mean age of the majority of cases was around 30 y (range, 20-40 y).^12^ Although in recent years, numerous cases of alcohol poisoning cases have been reported, eg, the 2004 Shiraz incidents (62 morbidity and 11 mortality cases), or those of Rafsanjan in 2013 (694 morbidity and 6 mortality cases),^3,4^ the early 2020 event occurred in a shorter period and had a higher number of affected people (more than 6150 cases with 804 deaths).

## Discussion

Preliminary investigations into the news and media showed that the false propaganda of racketeers misled the public into believing that, because alcohol can remove the viruses, its consumption can destroy the coronaviruses having entered the body. This myth motivated the frightened youth to consume alcohol, which led to an unexpected rate of alcohol poisoning. Based on the investigations, due to the common restrictions on alcohol production and sales in Iran, the available inexpensive alcohol is illegitimately produced in nonstandard conditions, and thanks to the present health crisis, the swindlers have found the opportunities to distribute their products with the logo of well-known brands. Some young people, not knowing the real nature of most of the available bottle alcohol variety, attempt to buy and use them due to their low prices.^13^

**Figure 1.**
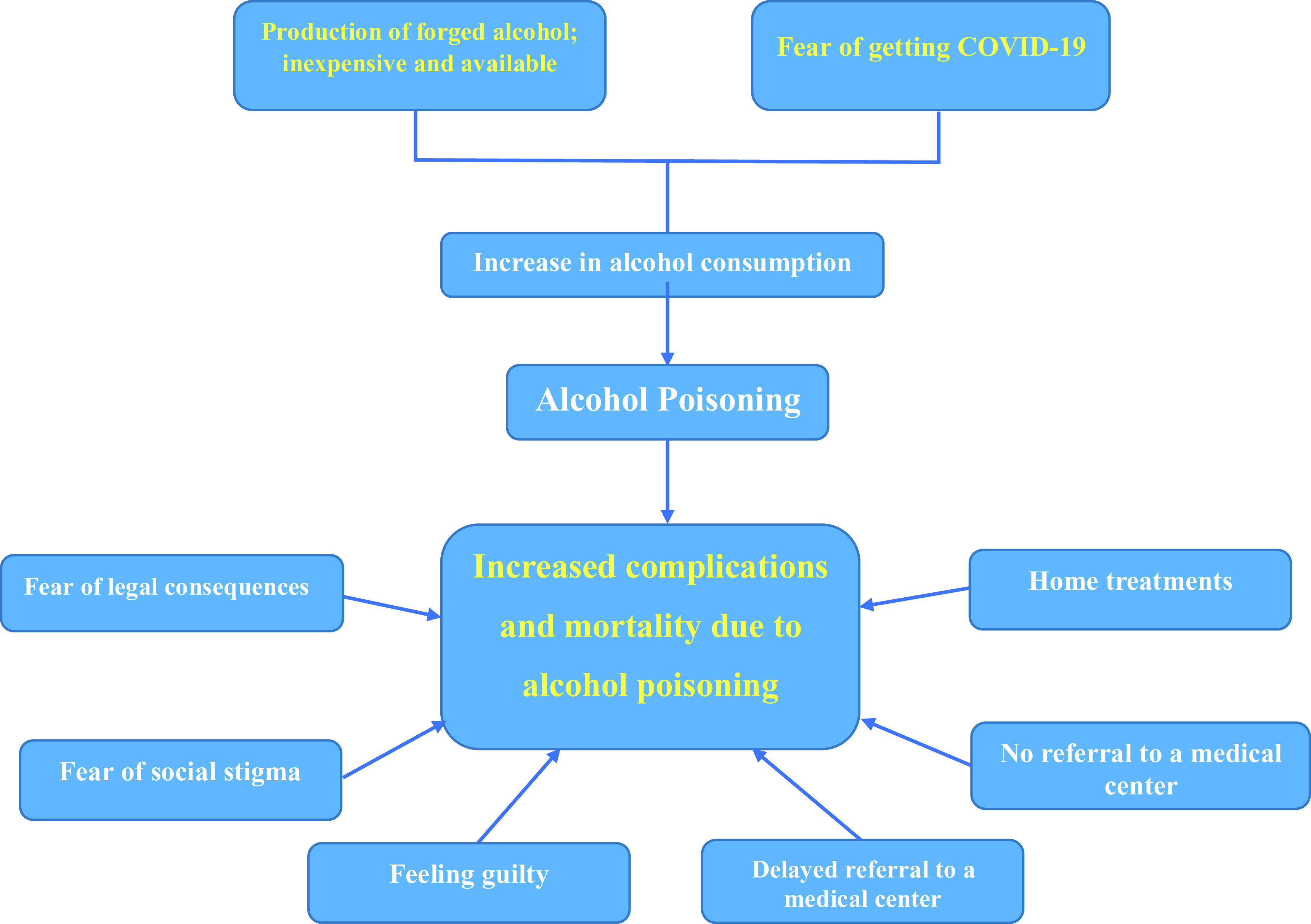
Factors causing and aggravating alcohol poisoning in Iran at the onset of the COVID-19 pandemic.

Generally, the consumption of alcoholic beverages has increased among Iranian youth. As these drinks, too, are being prepared illegally in the country (containing around 40% of alcohol compared with 3% in Europe),^14^ they have significantly increased the side-effects and mortality rates associated with alcohol poisoning. Despite the issues mentioned above, Iran, altogether, still enjoys an optimal state in terms of alcohol addiction; however, because no clear statistics are available, one cannot be hopeful that this optimal state would persist.^15^ The complication is, therefore, not with the addiction but with the inappropriate and unsafe consumption of alcohol. It is an undeniable problem that leads to many social pathologies.^14^ As a social pathology, alcohol consumption and abuse has a complex economic, social, and cultural causal network, and imposes a heavy security and health-related burden on the society.^15^ Haghdoost et al. examined the causes of alcohol consumption after the poisoning epidemics in Rafsanjan in 2013. They reported that the main causes of alcohol consumption among the youth were, namely, lack of awareness, inaccurate attitudes and beliefs, a change in values with regard to alcohol consumption, social networks supporting alcohol consumption, environmental and background factors, and the managerial and governmental structures^15^ (Figure 1).

Delirrad and Mohammadi, while approving the above-mentioned ideas with respect to the public’s unawareness and erroneous beliefs regarding alcohol as a protective agent, argue that at the beginning of COVID-19 outbreak, a variety of recommendations spread by means of social media, such as the idea that gurgling or drinking alcoholic beverages would disinfect the mouth or inside of the body and prevent the infection from entering the body. These false beliefs led to numerous methanol poisoning blindness and deaths. These authors further state that, in Iran, the majority of hospitals cannot determine blood concentration of toxic alcohol nor the required antidotes and medications for the treatment of intoxicated patients due to lack of proper laboratory equipment. In this study, the ruling authorities have been considered responsible for all aspects of this dilemma.^7^ Figure 1 represents some underlying factors found in the investigation.

## Conclusions

It seems that alcohol consumption should not be viewed only as legal and cultural issues, but as a social pathology that requires social and psychological solutions. According to the studies, many people do not know adequately about the different types of alcohol and their side-effects.^15^ On the other hand, in Iran, fear of legal consequences, feeling guilty,^16,17^ and social stigma either hinders those affected by alcohol poisoning from visiting the health-care centers or causes them to have significant delays in visiting these centers. Inappropriate home treatments have intensified the situation as well.^13,18^ These reasons have increased the side-effects and mortality rates associated with alcohol poisoning.^13^ However, the same restrictions may deter the related authorities from attempting to raise the awareness of the public, especially the youth, about the side-effects of methanol consumption.^19^

At the time of the current COVID-19 pandemics, which has become the main concern of all societies, people have become more sensitive to health news and recommendations. It is, therefore, mandatory that the previous considerations be put aside, and the side-effects of alcohol consumption be clearly and openly discussed with the people. To achieve this end, all media, social networks, and peer groups must be exploited to raise social awareness by providing them with sustainable and accurate information coming from credible sources. Measures must be taken to increase people’s trust in health-care recommendations so that they would not be misled by false news and rumors. It must be specified that “alcohol consumption will not help prevent COVID-19,” while COVID-19 epidemics can be controlled by adherence to primary health principles and stopping the transmission cycle.
